# From External to Internal: The Mediating Role of Educational Aspiration and Depression on the Association Between Caregiver Expectation and Academic Performance Among Chinese Rural Students

**DOI:** 10.3390/bs15050698

**Published:** 2025-05-19

**Authors:** Jia Zhuang, Yuying Tong

**Affiliations:** 1 Department of Applied Social Sciences, The Hong Kong Polytechnic University, Hong Kong; 2 Department of Sociology, The Chinese University of Hong Kong, Hong Kong

**Keywords:** academic performance, caregiver expectation, depression, educational aspiration, Chinese rural students

## Abstract

Although empirical studies have consistently suggested that educational expectations from caregivers would shape students’ internal factors of learning (e.g., educational aspirations, mental status, and motivation), how such processes further link with students’ academic ability received relatively little scholarly attention. Using the longitudinal China Family Panel Studies survey data, this paper applies structural equation modelling to examine the interrelationship between caregiver educational expectation, educational aspiration, depression, and academic performance of Chinese rural students (*N* = 584). The results show a negative correlation between caregiver educational expectation and rural students’ depression. Further, we find that when family wealth, fathers’ education level, students’ age, gender, and school type (public/private) are controlled for, caregiver educational expectation positively correlates with rural students’ educational aspiration and negatively associates with their depression, which is further positively and negatively associated with their academic performance, respectively. Our findings indicate that caregiver educational expectations might play a salient role in intervention programs enhancing rural students’ academic performance through promoting their educational aspirations and alleviating their depression. The results of this study offer fresh insight into how rural educators, families, and practitioners might address the issue of Chinese rural students’ low academic outcomes.

## 1. Introduction

Educational development is a critical aspect of children’s growth. It is a global pledge of equitable cultivation of adolescents from different cultural, ethnic, and socio-economic backgrounds. In China, the rapid economic growth is accompanied by a substantial rural–urban disparity in students’ educational achievement (X. Wu, 2024; Y. Lu et al., 2019). The Report on the State of Children in China by L. J. Chen et al. (2015), for example, reveals a significant gap between rural and urban children aged 0–15 in their academic achievement, socio-emotional wellbeing, and cognitive growth. Another study by Hao and Yu (2017) also found that the cognitive ability of urban ninth graders is 24.8% of a standard deviation above their rural peers. Likewise, the recent study by Xiang and Stillwell (2023) adopted a new analytical approach based on Amartya Sen’s capability theory and found that the overall rural–urban attainment gap reflects the historical rural–urban socio-economic development differences of the nation. They noticed that the educational disparity between rural and urban students remained stable from 2000 to 2010.

While previous scholarly efforts have recognized that Chinese rural students’ educational development is challenged by multifarious and interrelated familial socio-economic factors, more recent studies have been taking a strength-based approach to investigate the protective and promotive factors and mechanisms contributing to rural students’ positive educational development in spite of the underprivileged rural conditions (e.g., L. Li & Liu, 2022; B. Wang et al., 2022; Xu & Montgomery, 2021). One of the often discussed factors is caregiver educational expectation, which was found to be high among Chinese rural parents and grandparents of diverse socioeconomic backgrounds owing to their high value of education rooted in their Confucius cultural beliefs (Jin, 2024; Kung & Lee, 2016; Tan, 2017). Among global studies, caregiver educational expectations have been documented to be a salient factor in shaping students’ academic performance and educational attainment (X. Fan & Chen, 2001; Yamamoto & Holloway, 2010; T. Zhang & Huang, 2022). For instance, the random-effects meta-analysis of Pinquart and Ebeling (2020) examined 169 relevant studies around the world and identified both cross-sectional and longitudinal associations between caregiver educational expectation and students’ academic performance after controlling family socioeconomic status. In China, a study found that educational expectation from parents partially mediates the association between family assets and their academic performance (Fang et al., 2020).

From a family system perspective, the reasons behind the positive correlation between caregiver educational expectation and the academic performance of children are mainly twofold. First, high and positive caregiver expectations affect adolescents’ external learning environment (e.g., home-based parental involvement and teacher support), which in turn, shapes their schooling and educational experience (Bashir & Kaur, 2017; T. Zhang & Huang, 2022). The other line of reasoning lies in the mediating effect of rural children’s internal learning-related characteristics on the association between caregiver expectations and their academic performance. Specifically, high and positive educational expectation from caregivers positively influence students’ internal learning qualities, which further contribute to their positive learning outcomes. The internal learning qualities have been identified by studies worldwide as students’ educational aspiration (X. Chen et al., 2023; Xu & Montgomery, 2021), academic self-efficacy (Neuenschwander et al., 2007), intrinsic motivation (Yamamoto & Holloway, 2010), mental status or academic anxiety (Mirawdali et al., 2018), etc.

To date, while much scholarly effort has been paid to investigate the influence of caregiver expectation on students’ external learning environment, less attention has been paid to how students’ internal factors mediate the relationship between caregiver expectations and educational outcomes. The present study aims to fill this knowledge gap by investigating the mediating role of educational aspiration and depression in the relationship between caregiver educational expectations and academic performance among Chinese rural students. Regarding educational aspiration, the Wisconsin Model of Status Attainment has depicted a pattern of intergenerational transmission of educational values and attitudes in determining an individual’s educational and occupational attainment. Caregivers’ educational expectations for their children can be passed down and translated into their kids’ educational aspirations, which further shape their educational achievement. As for depression, adolescents undergo significant physical, cognitive, emotional, and social changes (Graber et al., 2018; Lerner & Foch, 2021), making them more sensitive to emotional and behavioral disorders that can hinder learning (Blum et al., 2014; Eccles et al., 2003). Effective educational development during this period requires external support that aligns with their evolving psychological needs (Braddock & McPartland, 1993). However, rural students often receive inadequate psychological support due to factors like separation from migrant parents, low socioeconomic status, limited school resources, and insufficient involvement from caregivers (Cui et al., 2021; Xie et al., 2019). This lack of support poses considerable challenges to the mental well-being and educational progress of Chinese rural adolescents (Davidson & Adams, 2013). Nevertheless, positive expectations from caregivers might provide essential familial warmth, serving as a compensatory factor for students facing multiple adaptive challenges (Davidson & Adams, 2013; Simpkins et al., 2015). Drawing upon the longitudinal China Family Panel Studies (CFPS) survey data, this study examines the mediating role of depression and educational aspiration on the relationship between caregiver educational expectation and academic performance among Chinese rural students.

## 2. Theoretical Background

### 2.1. The Mediating Effect of Educational Aspiration Between Caregiver Expectation and Academic Performance

Educational aspiration is students’ expectations of academic success and the demonstration of educational goals and orientations (Loh, 2019). The discussions regarding the positive correlation between caregiver expectation and adolescents’ educational aspirations were based on the Status Attainment Theory, which accentuates the influence of external factors on students’ aspirations (Sewell et al., 1970). The Wisconsin model of status attainment specified the mediation mechanism of individual socio-psychological qualities—educational aspiration, in particular—in the relationship between family and school factors and youth educational and occupational attainment (Kristensen et al., 2009). Perceived caregiver educational attitudes, along with encouragement from family and schools, shape individual students’ learning beliefs and status aspirations. Caregivers serve as ‘expectancy socializers’ and role models for children in their proximal process (Rumberger, 1983). They convey their recognized attitudes and values to their children and help them establish their own belief systems through family communications.

**Figure 1:**
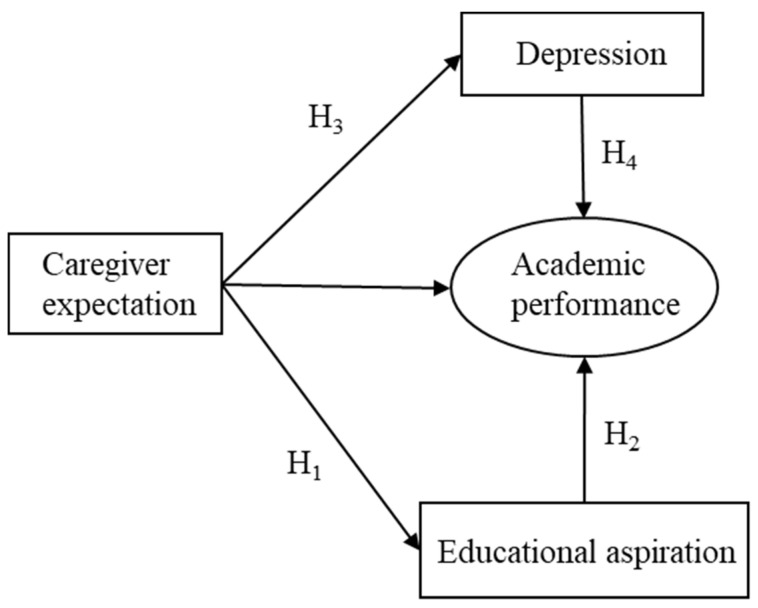
The Conceptual Model of the Mediating Effect of Depression and Educational Aspiration on the Relationship Between Caregiver Expectation and Academic Performance.

Aside from the Status Attainment Theory, one common theoretical tenet emphasized in Albert Bandura’s Social-cognitive Theory and Lev Vygotsky’s Sociocultural Theory of Cognitive Development is the pivotal role played by family socialization in forming the characteristics and personality of a child (Bandura, 2014; Gauvain, 2001). Observations and perceptions from social interactions with significant others are integral to adolescents’ cognitive knowledge and skill acquisition (Froiland et al., 2013). In effect, adolescents immersed in high caregiver expectations always exhibit high academic aspirations because the positive attitude and value toward education could be transmitted through generations (Goings & Shi, 2018). Perception of high expectations from parents constructs adolescents’ value of education and notion of academic success (Nihal Lindberg et al., 2019). International studies also revealed that perceived parental expectations augment students’ executive functions, nurture their self-concept, boost their sense of self-mastery in learning, and sharpen their problem-solving skills (Assari, 2018; Flouri & Hawkes, 2008; Ma et al., 2018). In this vein, we propose the following hypothesis (see Figure 1):

**Hypothesis** **1** **(H_1_).** *Caregiver educational expectation would be positively associated with rural students’ educational aspiration.*

In addition, educational aspiration is a crucial internal asset leading to positive youth educational development (Buchmann et al., 2022). Such a correlation is attributed to students’ positive attitudes toward formal schooling, which directly affects individuals’ academic performance. Students who aspire to achieve high academically exhibit a strong sense of purpose in education, a high intrinsic academic motivation and enthusiasm, and academic self-efficacy (Masten & Coatsworth, 1998). The motivation theory suggests that children with high extrinsic and intrinsic motivations are more likely to value their educational experience and place great importance on education (W. Fan & Wolters, 2014). In addition, the possible-selves perspective suggests that high educational aspirations lead to a positive self-perception, which in turn drives students to take goal-directed actions in line with their imagined self (Oyserman et al., 2007). The positive posture toward education not only leads to positive academic scores but also to higher educational qualifications, since students’ educational aspirations drive them to make extra effort in their learning and prevent them from early school dropouts (Wigfield & Eccles, 2002). Thus, we propose the following hypothesis (see Figure 1):

**Hypothesis** **2** **(H_2_).** *Rural students’ educational aspiration would be positively correlated with their academic performance.*

Considering H_1_ and H_2_ as a whole, it is hypothesized that students’ educational aspirations would mediate the association between their caregiver expectations and academic performance.

### 2.2. The Mediating Effect of Depression Between Caregiver Educational Expectation and Academic Performance

Apart from motivating children via their own educational aspirations, adolescents’ depression may also act as a mediating factor influencing their academic performance. While there is hardly any debate among educational researchers regarding the positive association between caregiver expectation and adolescents’ aspirations, the existing evidence based on prior literature is mixed as to how caregiver expectations affect adolescents’ mental health. On the one hand, adolescents unable to live up to their caregiver expectations suffer from mental pressure and depression (DiBartolo & Rendón, 2012). Likewise, students whose self-aspiration mismatches with their caregivers’ expectations are confronted with multiple stressors and depressive symptoms (Rutherford, 2015).

On the other hand, Bornstein’s (2013) cross-cultural study underlines that parents’ expectations determine their childrearing attitude and practice, which affects the early life experiences and mental health of the child. Caregivers’ positive expectations are reflected in their concern, attention, and family investment in their children’s growth (Hao & Yeung, 2015). Caregivers who maintain high expectations have a strong motivation to devote time and effort to family communication, which would deliver parental warmth to their child and help establish a positive parent–child relationship (Simpkins et al., 2015). Such a relationship is associated with a wide range of positive psychological outcomes in adolescents, including less internalizing and externalizing problems and higher psychosocial competence (Steinberg, 2001).

In China, preschoolers who receive close attention from their caregivers demonstrate high social and emotional competence (Ren & Pope Edwards, 2015). Such correlation is mediated by parenting style. Parents with high expectations were likely to practice authoritative parenting and were more caring and responsive to their children’s social and emotional growth. Another study using the longitudinal data from the China Education Panel Survey reveals that parental expectation positively affects Chinese adolescents’ subjective psychological wellbeing (H. Lu et al., 2021). The correlation is mediated by family learning resources and parent–child relationships. Students with high parental expectations obtained sufficient tender solicitude from parents and embraced positive family relations (Gauvain et al., 2013). These proactive parental engagements are believed to be beneficial to adolescents’ psychological wellbeing. We thus proposed the following hypothesis (see Figure 1):

**Hypothesis** **3** **(H_3_).** *Caregiver educational expectation is negatively correlated with Chinese rural students’ depression.*

As regards the relationship between depression and adolescents’ educational development, scholarly evidence abounds in explaining the negative effect of depression on adolescents’ academic performance (L. Zhang & Yang, 2019). First, depression in adolescence is always fraught with broad attentional problems and motivation challenges (Gotlib & Joormann, 2010). Reduced motivation might undermine students’ effort in learning and make it difficult for them to engage in learning activities (Scheurich et al., 2008). For example, studies with Hong Kong primary and secondary school students revealed a positive association between depression and low self-efficacy and learning motivation (Chang et al., 2018). Likewise, prolonged difficulties with concentration could interfere with adolescents’ information acquisition ability and knowledge consolidation processes (Kovacs & Goldston, 1991). Further, school students with depression are inclined to demonstrate low self-esteem, poor initiative, and a sense of worthlessness (Beck & Alford, 2009). They tend to experience social withdrawal and difficulties in social relationships in school settings, which might further negatively affect their learning experience and performance (Fröjd et al., 2008). Taken together, we propose the following hypothesis (see Figure 1):

**Hypothesis** **4** **(H_4_).** *Depression would be negatively associated with academic performance among Chinese rural students.*

Considering H_3_ and H_4_ as a whole, it is hypothesized that students’ depression would mediate the association between their caregiver expectation and academic performance.

## 3. Materials and Methods

### 3.1. Data and Participants

This study utilizes longitudinal data from the China Family Panel Studies survey to examine the relationship between caregiver expectations and the academic performance of rural Chinese adolescents. Launched in 2010 by the Institute of Social Science Survey of Peking University, the CFPS survey collects comprehensive information about individuals and families regarding their economic activities, educational development, mobility, health, etc. Since 2010, using Probability-Proportional-to-Size sampling, longitudinal data were collected biennially from 25 provinces/cities/autonomous regions, which together contain 95% of the national population (Q. Wu et al., 2021). Both socioeconomic status and administrative units were applied as the stratification variables. The primary, second-stage, and third-stage sampling units are the administrative districts/counties, administrative villages/neighborhoods, and households, respectively. A systematic selection was applied in the third stage with a random starting point and equal probability method.

The CFPS 2020 was conducted online due to the pandemic, and the cognitive ability test was excluded. We then used the latest panel data collected in 2018 (Time 2) and 2016 (Time 1). The 2016 survey was conducted from July 2016 to May 2017. It collects information from 14,700 households and 45,300 individuals. Among them, 2560 are children aged from 10 to 15, consisting of 1525 and 1035 from rural and urban areas, respectively. The 2018 survey was conducted from June 2018 to May 2019. Questionnaires were collected from 15,000 families and 44,000 individuals. In total, 2607 participants aged 10 to 15 completed the self-questionnaire, while another 2827 child reports were collected from their proxies. We merged the individual and proxy datasets and obtained 2473 combined early adolescent questionnaires provided by the children and their guardians. Among them, 1419 cases are from rural areas. Yet, 22% of the CFPS questionnaires (2018 wave) were obtained through phone interviews, in which the cognitive test was excluded (Q. Wu et al., 2021). The number of available observations from the 2018 survey is 1308 after removing 12 missing cases and 149 observations without a cognitive score. We combined the 2018 person dataset with the 2016 child dataset to obtain the independent variable (i.e., caregiver expectation in 2016) and the baseline measures (i.e., depression and educational aspiration in 2016) for our analysis. We then merge the combined dataset with the 2016 person dataset and family economy dataset to incorporate the covariates of the father’s educational level and family income, respectively. The final sample size for mediation analysis is 584.

Table 1 details the demographic profile of our participants. While the distribution of participants in each age group is nearly even, this study includes a slightly bigger proportion of male participants (56%). When surveyed in 2018, more adolescents were pursuing junior secondary education (57%) than primary (42%) and senior secondary education (2%). The majority (89%) of them were attending public schools.

### 3.2. Measures

*Caregiver expectation*. In CFPS, child proxy questionnaires were completed by parents and grandparents who took care of the child. They are the most proximal family members to the child (Q. Wu et al., 2021). In the CFPS 2016 survey, caregiver expectation is assessed by one question: What is the average score out of a total of 100 that you expect your child to achieve in this/next semester? The answers to the question range between 0 and 100. A higher score indicates a higher caregiver educational expectation. This measure is also preferred by H. Li and Yeung (2019) in their investigation of the relationship between family factors and the academic resilience of Chinese rural students.

*Depression*. The Center for Epidemiologic Studies Depression Scale (CES-D) was widely applied by international studies to measure individuals’ depression. It is worth noting that depression measured by CES-D does not match the clinical definition of major depression disorder or episodes diagnosed based on the DSM-IV code table (Qin et al., 2018). Instead, it indicates an individual’s frequency of depressive symptoms within a certain period. The scale consists of 20 self-evaluation items. Each item is a short statement about the emotional, cognitive, and behavior-related components of depression. In its 2016 survey, CFPS randomly selected eight items from the pool and generated an abbreviated version of CES-D (Q. Wu et al., 2021). The abbreviated version was also utilized in the 2018 survey. The items were evaluated on a four-point Likert scale in relation to the frequency of depressive symptoms experienced during the foregoing week. Examples of the eight items are “I felt depressed” and “My sleep was restless”. Each item is scored from 1 (almost never; less than one day) to 4 (most of the time; 5–7 days). Two items are worded in a positive direction to reduce the response bias. After the reverse, the accumulative scores range from 8 to 32. A lower score indicates less presence of symptomatology. The Cronbach’s alpha of this scale was 0.684, which is considered acceptable for a scale with a small number of items.

*Educational aspiration*. Adolescents’ educational aspiration is assessed by the extent to which individuals want to achieve in school. In the CFPS 2018 survey, adolescents’ educational aspiration was measured with one item (what is the minimum level of education you think you should obtain?). Response categories consist of “no need to go to school”, “primary school”, “Junior high school”, “senior high school”, “3-year college”, “4-year college/Bachelor’s degree”, “Master’s degree”, and “Doctoral degree”. Responses were continuously coded from 1 (no need to go to school) to 8 (Doctoral degree). A higher score suggests a higher educational aspiration.

*Academic performance*. CFPS performed two different sets of cognitive tests in 2016 and 2018. The academic performance test of this analysis is adopted from the cognitive test of CFPS 2018, which consists of word and math tests. According to CFPS, while the 2016 cognitive test represents respondents’ potential intelligence, the word and math test scores of its 2018 cognitive ability test reflect participants’ academic performance (China Family Panel Studies, 2022). This measure of academic performance has been applied in empirical studies (e.g., Tong et al., 2021; A. Li, 2019). The design of the two tests is based on the Guttman Scale, which positions test items drawn from the standard curriculums in primary and secondary schools in order from easy to hard. The word test includes 34 Chinese characters. The math test incorporates 24 mathematical calculations, including arithmetic, exponents, etc. The score of academic performance ranges from 0 to 58. A higher score indicates a higher academic performance.

*Control variables*. Baseline controls were included for the two mediators (i.e., depression and educational aspiration in 2016). In addition, we controlled for age, gender (0 = female, 1 = male), school type (0 = private school, 1 = public school), parental time (average times per week for a child being with his/her parent), father’s educational attainment, and family wealth.

### 3.3. Analytic Approach

The means and standard deviations of each variable were calculated using Stata 17.0 software, followed by the analysis of the interrelations of variables. To test the mediating effect of depression and educational aspiration, structural equation modelling (SEM) was instructed in Stata under maximum likelihood estimation. In the constructed mediation model, caregiver educational expectation was entered as the predicting variable, while child academic performance was the outcome variable that was also set as a latent variable inferred from word and literacy scores. Depression and student educational aspiration were entered as two mediating variables. Baseline controls were added for the two mediators. In addition, adolescents’ sex, age, school type, parental time, family income, and their father’s education level are reported to be associated with their depression, educational aspiration, and academic performance. They are controlled in the model. The calculation and estimation of indirect and total effects are performed. A 95% significance level is applied for hypothesis testing and confidence interval estimation. Measurement and structural models are deemed to fit the data when the chi-square p-value is below 0.05, the root mean square error approximation (RMSEA) estimate is less than 0.08, the standardized root mean square residual (SRMR) is below 0.05, and the comparative fit index (CFI) reports is above 0.95 (Hu & Bentler, 1995).

## 4. Results

### 4.1. Descriptive Statistics

Descriptive statistics and correlations among variables are shown in Table 2. Rural students’ academic performance at Time 2 positively correlates with caregiver expectation (*r* = 0.17) at Time 2, students’ educational aspiration (*r* = 0.21) at Time 2, and father’s education level (*r* = 0.23) at Time 1, while negatively correlates with their depression (*r* = −0.13) at Time 1. Caregiver expectation at Time 1 is negatively associated with adolescents’ depression (*r* = −0.11) and positively correlates with their educational aspirations (*r* = 0.14) at Time 2. Rural adolescents’ depression is negatively correlated with their educational aspirations (*r* = −0.09), both at Time 2.

### 4.2. SEM Analysis

**Figure 2:**
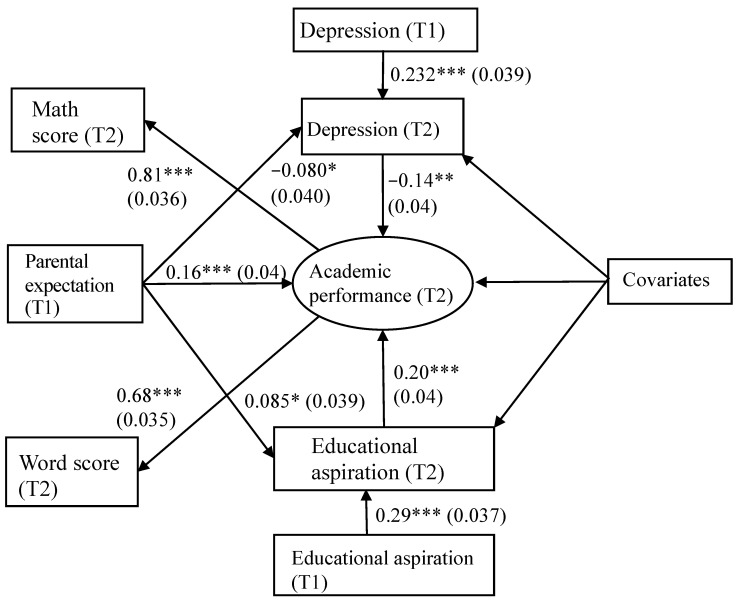
Mediation Model Testing the Mediating Effect of Depression and Educational Aspiration on the Relationships Between Caregiver Expectation and Academic Performance. *Note*. Standardized coefficient; standard errors in parentheses; (* *p* < 0.05; ** *p* < 0.01; *** *p* < 0.001); covariates include age, gender, school type, parental time, father’s educational level, and family wealth.

Figure 2 shows the empirical estimations of the model. The fitness of the model is adequately acceptable (χ^2^ (15) = 28.65, *p* = 0.018; RMSEA = 0.040; CFI = 0.973; SRMSR = 0.021; TLI = 0.924). Table 3 presents the unstandardized coefficients of the SEM model paths. The model shows that, when students’ age, gender, attending school, father’s educational level, and family wealth are controlled, higher caregiver expectations lead to higher educational aspiration (*β* = 0.085, *p* = 0.030), supporting H_1_. In addition, while rural students with higher educational aspiration performed better on the math and word test (*β* = 0.119, *p* < 0.000), depression hampers their academic performance (*β* = −0.145, *p* = 0.001), supporting H_2_ and H_4_. Further, the results demonstrate that caregiver expectation is negatively associated with Chinese rural adolescents’ depression (*β* = −0.080, *p* = 0.046), supporting H_3_. These results confirm that both depression and educational aspiration mediate the relationship between caregiver expectation and students’ academic performance. The model explains 37% of the variation in rural students’ academic performance (*R*^2^ = 0.37).

Table 4 displays the direct, indirect, and total effects of different relationships in the model. The direct effect of caregiver expectation on the academic performance of early adolescents is statistically significant (direct effect = 0.054, 95% CI = 0.025 to 0.083). The indirect effect of caregiver educational expectation on academic performance as mediated by depression and educational aspiration is statistically significant (indirect effect = 0.010, 95% CI = 0.002 to 0.017). The indirect effect of caregiver expectation on academic performance via depression and students’ educational aspirations is 0.004 and 0.006, respectively. This implies that rural adolescents’ educational aspiration might play a more significant role in mediating the relationship between caregiver expectation and academic performance. The total effect of caregiver expectation on academic performance is statistically significant (total effect = 0.064, 95% CI = 0.034 to 0.093). The ratio of the indirect effect to the total effect is 0.156, implying that depression and educational aspiration explain around one-sixth of the effect caregiver expectation has on rural adolescents’ academic performance. Moreover, the direct relationship between caregiver expectation and academic performance remains significant after considering the two mediators, indicating that depression and educational aspiration function as partial mediations.

## 5. Discussion and Implications

Although extant literature has identified a positive correlation between caregiver expectation and youth academic performance, little scholarly effort has been invested in exploring the mechanisms underlying this relationship in the rural Chinese context. Using the CFPS data, the present study fills this knowledge gap by examining the roles of Chinese rural adolescents’ educational aspirations and depression in mediating the positive relationship between caregiver expectations and academic performance.

The investigation is distinctly significant among the Chinese rural student population for two reasons. First, extant studies concerning students’ external expectations are centered on parental expectation because parents are considered most proximal to a child (e.g., Zhao & Bodovski, 2021; Zhuang et al., 2024). In rural China, however, around one-third of its student population is left behind by one or both parents and residing with other caregivers (e.g., grandparents) (Chai et al., 2019). Even those physically living with parents might not receive much care from them because rural parents usually spend the majority of their time and energy on making ends meet and leaving their kids to their grandparents (Yu, 2020). Thus, given the salient role played by other caregivers rather than parents in Chinese rural students’ educational development, it is crucial to take their expectations into consideration and examine their impact on rural students’ academic performance. Second, educational resource scarcity in rural households and schools makes rural adolescents’ educational development an urgent issue at the national level. Given that rural caregivers’ concrete investments in their children’s education and schooling are confined by their deficiency of cultural, social, and economic capital to a certain extent, their educational expectations toward their rural kids serve as a crucial path to enhance their kids’ educational outcomes (Xu & Montgomery, 2021). Its mechanism deserves more scholarly attention, and such knowledge might provide practical insight for rural educational officials, stakeholders, school educators, and rural caregivers concerned with the academic development of Chinese rural adolescents in their parenting practice, intervention program design, and curriculum reform.

Our study yields a few important findings. First, rural adolescents’ educational aspirations mediate the relationship between caregiver educational expectations and their academic performance. This finding is consistent with the Status Attainment Theory (Bozick et al., 2010), which suggests that parents’ positive attitudes toward education can be transmitted across generations. Perceived high educational expectations from parents can be internalized by adolescents to build up their positive value of education. In the present study, Chinese rural adolescents whose caregivers hold high expectations of academic performance maintain a high aspiration for their future educational attainment. Adolescents’ perception of education determines their learning attitude and effort. Those who aspire to achieve high levels are likely to have strong learning motivation and put effort into their learning and development (Assari, 2018). When other individual factors are controlled for, adolescents with more motivation and action in learning are likely to have a better academic outcome. This study is consistent with the conclusions drawn by previous empirical studies based on the Wisconsin model of status attainment, which manifests the effect of educational aspiration in mediating caregiver influence (caregiver expectation in this study) and student achievement (Bozick et al., 2010).

Second, this study reveals that educational expectation from parents mitigates Chinese rural adolescents’ depression. It is also noted that caregiver educational expectations and rural adolescents’ educational aspirations are high in this study. It suggests that the mismatch between caregiver expectations and students’ aspirations, which harms rural students’ self-esteem and causes them severe academic anxiety and pressure, might be small among Chinese rural adolescents (Rutherford, 2015). Rural students in this study exhibit, on average, mild depression. Furthermore, students with depression generally perform poorly in their knowledge acquisition and consolidation (Scheurich et al., 2008). Therefore, we observe a negative relationship between depression and academic performance among rural adolescents in our analysis.

The results of this study offer fresh insight into how we might address the issue of Chinese rural adolescents’ low academic outcomes. While sufficient family involvement and economic support might not be realistic for rural parents who live apart from their children or hold no resources to support their children’s education, high caregiver educational expectations are possible resorts for them to alleviate depressive symptoms and increase the educational aspiration of their children. It is thus imperative for educators, rural school managers, and policymakers to implement effective intervention programs to promote rural parents’ expectations of their children when it comes to educational levels. More importantly, to optimize the treatment outcomes, these intervention programs should adopt a combined approach to involve both rural caregivers and adolescents and take into consideration not only caregiver educational expectations but adolescents’ own academic attitudes and mental health. On top of this, in addition to the traditional way of school counselling or attentive community support, we suggest that appropriate parental training should be delivered to rural caregivers to equip them with the skills of communicating their high educational expectations to their child to build up their positive attitudes towards education and improve their mental health.

While we believe that the findings of this nationally representative research advance our understanding of the complicated nexus between caregiver educational expectations and adolescents’ educational development, a few limitations regarding the research measures and conceptual framework should be noted. First, the construct of caregiver expectation was gauged with only one item. It might not fully capture the multi-dimensional nature of caregiver expectations. Further research should consider applying validated instruments in describing caregiver expectations, such as the Parental Expectation Scale by L. F. Wang and Heppner (2002). Similarly, given that there are tailor-made scales measuring students’ depression (Center for Epidemiological Studies Depression Scale for Children; CES-DC), it might be better for later studies concerned with youth depression and academic performance to utilize the modified version of CES-D for more precise and accurate measurement.

Second, the present study investigated the mediating mechanisms of adolescents’ depression and educational aspirations. However, as discussed in our literature review, caregiver expectation also affects students’ social competence, learning habits, self-efficacy, and internal locus of control. These variables exert a profound impact on adolescents’ learning and developmental outcomes. Some of the unexplained total effects of caregiver expectations on adolescents’ academic performance might be mediated by these variables. We suggest that future research should take these variables into consideration to enrich the picture of the relationship between caregiver expectations and adolescents’ academic performance.

## Figures and Tables

**Table 1:** Demographics of participants.

Demographic Characteristics	Frequency (*N* = 584)	Percentage (%)
Gender		
Female	258	44.18
Male	326	55.82
Age		
11	5	0.86
12	143	24.49
13	152	26.03
14	157	26.88
15	127	21.75
Educational level		
Primary	244	41.78
Junior high	331	56.68
Senior high	9	1.54
School type		
Public	519	88.87
Private	65	11.13
Caregiver		
Mother	303	51.88
Father	163	27.91
Others	118	20.21

**Table 2:** Means, standard deviations, and correlations among variables.

Variables	Min	Max	M	SD	1	2	3	4	5	6	7	8	9
1. Academic performance ^T2^	0	53	37.65	8.39	—								
2. Caregiver expectation ^T1^	4	100	90.3	10.87	0.17 **	—							
3. Depression ^T2^	8	26	11.89	2.96	−0.13 **	−0.11 *	—						
4. Educational aspiration ^T2^	3	9	6.1	1.24	0.21 ***	0.14 ***	−0.09 *	—					
5. Father education level ^T1^	1	6	2.35	0.97	0.23 ***	0.05	−0.03	0.10 *	—				
6. Family wealth (000) ^T1^	0.16	396	51.16	47.02	0.05	0.05	−0.04	0.03	0.20 ***	—			
7. Parental time ^T1^	0	24	16.34	8.01	−0.03	0.08 *	−0.03	0.08	0.01	0.01	—		
8. School type (public = 1) ^T1^	0	1	0.889	0.32	−0.09 *	−0.05	0.04	−0.02	−0.07	−0.10 *	0.12 **	—	
9. Age ^T1^	11	15	13.44	1.11	0.37 ***	−0.03	0.05	0.02	0.02	−0.03	−0.08 *	0	—
10. Gender (male = 1) ^T1^	0	1	0.56	0.5	−0.07	−0.02	−0.00	−0.12 **	−0.02	0.05	0	−0.12 **	−0.05

Note. ^T1^ refers to Time 1; ^T2^ refers to Time 2; (* *p* < 0.05; ** *p* < 0.01; *** *p* < 0.001).

**Table 3:** Results of SEM analysis.

	Coef. (*β*)	S.E.	*p*-Value	95% CI
Academic performance ^T2^				
Depression ^T2^	−0.145	0.042	0.001	[−0.226, −0.063]
Educational aspiration ^T2^	0.199	0.042	<0.001	[0.116, 0.281]
Caregiver expectation ^T1^	0.158	0.042	<0.001	[0.077, 0.240]
Parental time ^T1^	−0.022	0.042	0.599	[−0.105, 0.060]
Father education level ^T1^	0.223	0.042	<0.001	[0.140, 0.305]
Family wealth ^T1^	0.000	0.042	0.992	[−0.083, 0.084]
School type (public) ^T1^	−0.077	0.042	0.068	[−0.160, 0.006]
Gender (male) ^T1^	−0.002	0.044	0.896	[−0.087, 0.084]
Age ^T1^	0.442	0.038	<0.001	[0.368, 0.517]
Depression ^T2^				
Depression ^T1^	0.232	0.039	<0.001	[0.156, 0.308]
Caregiver expectation ^T1^	−0.080	0.040	0.046	[−0.159, 0.001]
Parental time ^T1^	−0.012	0.041	0.776	[−0.091, 0.068]
Father education level ^T1^	−0.024	0.041	0.559	[−0.104, −0.056]
Family wealth ^T1^	−0.028	0.041	0.491	[−0.109, 0.052]
School type (public) ^T1^	0.036	0.041	0.375	[−0.044, 0.116]
Gender (male) ^T1^	0.015	0.040	0.717	[−0.064, 0.094]
Age ^T1^	0.046	0.040	0.251	[−0.032, 0.124]
Educational aspiration ^T2^				
Educational aspiration ^T1^	0.294	0.037	<0.001	[0.221, 0.367]
Caregiver expectation ^T1^	0.085	0.039	0.030	[0.008, 0.161]
Parental time ^T1^	0.077	0.039	0.049	[0.000, 0.153]
Father education level ^T1^	0.060	0.040	0.131	[−0.018, 0.137]
Family wealth ^T1^	0.017	0.040	0.675	[−0.061, 0.094]
School type (public) ^T1^	−0.014	0.040	0.717	[−0.092, 0.063]
Gender (male) ^T1^	−0.109	0.039	0.005	[−0.184, −0.033]
Age ^T1^	0.050	0.039	0.199	[−0.026, 0.126]

Notes. ^T1^ refers to Time 1; ^T2^ refers to Time 2. Listwise deletion was used for this analysis.

**Table 4:** Direct, Indirect, and Total Effects Among Parental Expectation, Depression, Educational Expectation, and Academic Performance.

Relationships	Direct (95% CI)	Indirect (95% CI)	Total (95% CI)
1. Caregiver expectation ^T1^ -> Depression ^T2^	−0.022 (−0.043, −0.000)		−0.022 (−0.043, −0.000)
2. Caregiver expectation ^T1^ -> Educational aspiration ^T2^	0.010 (0.001, 0.018)		0.010 (0.001, 0.018)
3. Depression ^T2^ -> Academic performance ^T2^	−0.181 (−0.285, −0.077)		0.181 (−0.285, −0.077)
4. Educational aspiration ^T2^ -> Academic performance ^T2^	0.592 (0.337, 0.847)		0.592 (0.337, 0.847)
5. Caregiver expectation ^T1^ -> Academic performance ^T2^	0.054 (0.025, 0.083)	0.010 (0.002, 0.017)	0.064 (0.034, 0.093)

Notes. ^T1^ refers to Time 1; ^T2^ refers to Time 2.

## Data Availability

The data that support the findings of this study are openly available in China Family Panel Studies at https://www.isss.pku.edu.cn/cfps/ (accessed on 6 June 2023).
